# Poly(vinyl alcohol)/Gentamicin and Poly(vinyl alcohol)/Chitosan/Gentamicin: Promising Materials for Rapid Burn Wound Healing

**DOI:** 10.3390/gels11050352

**Published:** 2025-05-10

**Authors:** Anja Nikolić, Ivan Milošević, Ana Janković, Bogomir Bolka Prokić, Emilija Nićković, Danica Marković, Milena Stevanović, Maja Vukašinović-Sekulić, Vesna Mišković-Stanković, Tijana Lužajić Božinovski

**Affiliations:** 1 Department of Histology and Embryology, Faculty of Veterinary Medicine, University of Belgrade, Bulevar Oslobodjenja 18, 11000 Belgrade, Serbia; anja.nikolic@vet.bg.ac.rs (A.N.); nickovic.emilija@gmail.com (E.N.); danica@vet.bg.ac.rs (D.M.); ticavet@vet.bg.ac.rs (T.L.B.); 2 Innovation Center of the Faculty of Technology and Metallurgy, University of Belgrade, Karnegijeva 4, 11000 Belgrade, Serbia; mstevanovic@tmf.bg.ac.rs; 3 Department of Surgery, Orthopedy and Ophthalmology, Faculty of Veterinary Medicine, University of Belgrade, Bulevar Oslobodjenja 18, 11000 Belgrade, Serbia; bbprokic@gmail.com; 4 Department of Biochemical Engineering and Biotechnology, Faculty of Technology and Metallurgy, University of Belgrade, Karnegijeva 4, 11000 Belgrade, Serbia; vukasinovic@tmf.bg.ac.rs; 5 Faculty of Ecology and Environmental Protection, University Union-Nikola Tesla, Cara Dušana, 11000 Belgrade, Serbia; vesna.miskovicstankovic@unionnikolatesla.edu.rs

**Keywords:** chitosan, healing, in vitro, in vivo, wound dressing, rat

## Abstract

Scar formation and delayed wound healing pose significant challenges in treating skin injuries, especially in severe cases like burns and diabetic wounds. This study investigates the effectiveness of novel Poly(vinyl alcohol) (PVA)/Gentamicin (Gent) and PVA/Chitosan (CHI)/Gent hydrogels in promoting healing of second-degree burn wounds in a rat model. Following in vitro testing, these hydrogels were deemed non-toxic and suitable for in vivo analysis. Clinical evaluations were conducted on the 3rd, 7th, 14th, and 21st post-injury days, assessing parameters such as blistering, edema, redness, crust, bleeding, secretion, scar tissue formation, and wound contraction percentage. Histological analyses focused on re-epithelization and dermal evaluation at specific time points. Results showed that both hydrogels significantly reduced inflammation, particularly redness, by the 14th day and improved re-epithelization, with the PVA/CHI/Gent group outperforming on the 14th day and the PVA/Gent group excelling on the 21st day. Histological findings indicated increased fibroblast proliferation and collagen deposition in treated groups, suggesting enhanced dermal healing. The PVA/CHI/Gent hydrogel demonstrated notable antibacterial properties, likely due to the synergistic effects of CHI and Gent, leading to reduced inflammation and edema. Overall, both hydrogels show promise as effective wound dressings, facilitating faster healing and improved tissue recovery in burn injuries. This study supports the use of biomimetic scaffolds for enhanced wound management in clinical practices.

## 1. Introduction

Scar formation and delayed wound healing are significant challenges in treating skin tissue trauma [1,2]. Wound healing and tissue regeneration are dynamic physiological processes influenced by the interactions of the extracellular matrix, various cell types, and growth factors [3]. In severe conditions like trauma, diabetic, or burn wounds, the normal processes of re-epithelialization and dermal repair may be insufficient for healing [4]. Debridement, pressure reduction, and infection prevention are traditional wound treatment methods [5]. While effective for most injuries, larger skin wounds may need dressings that mimic skin tissue to support cell adhesion, migration, proliferation, and tissue generation by mimicking the extracellular matrix (ECM) [6]. Recent advancements in biomimetic scaffolds utilizing stem cells and bioactive substrates have created promising therapeutic options for treating full-thickness skin injuries [7]. Various materials and structures, such as hydrogels, films, and foams, are used as scaffolds to enhance healing and protect the skin from additional damage because they possess ECM-like structure, high porosity, and permeability [6]. Hydrogels are regarded as ideal skin substitutes due to their moisture retention, effective fluid absorbance, and high water retention capacity [8,9]. Their porous structure provides support in absorbing wound exudate, minimizing infection risk, and promoting an environment conducive to wound healing [8,9]. Additionally, utilization of natural-synthetic polymer composites in hydrogel fabrication results in a porous structure that benefits from the biocompatibility of natural polymers and the customizable properties of synthetic polymers [10].

Poly(vinyl alcohol) (PVA) is a synthetic polymer widely used in tissue engineering due to its excellent biocompatibility, biodegradability, non-toxicity, non-carcinogenicity, and water solubility [11]. This cost-effective polymer possesses favorable physical properties, including good transparency, low interfacial tension, and a high swelling ratio. To improve mechanical properties, PVA has often been combined with natural polymers, as is the PVA/chitosan composite hydrogel, with enhanced softness and flexibility [10,12]. Yang et al. [13] found that glycerol-modified PVA/chitosan hydrogels, created via irradiation and freeze-thawing, accelerated wound healing in rats. The hydrogel was non-toxic to fibroblasts and promoted epidermal maturation. Sung et al. [14] showed minocycline-loaded PVA/chitosan films enhanced wound closure in rats versus control sterile gauze, suggesting effective wound dressing potential.

Chitosan (CHI), a polysaccharide-based polymer, is particularly effective in wound healing due to its ability to reduce pain by blocking nerve endings [12]. It accelerates natural blood clot formation and minimizes scarring [15]. CHI-based hydrogels possess excellent bio adhesive properties, prevent microbial penetration, control inflammation, and enhance natural hyaluronic acid levels at the wound site [16,17]. Structurally similar to glycosaminoglycans in the ECM, CHI boosts fibroblast activation, regulates collagen fiber density and distribution, and promotes cell migration, granulation tissue formation, and vascularization- essential processes in wound healing [15,17]. Chitosan degradation by enzymes like lysozyme into chito-oligomers further enhances healing by activating macrophages and accelerating collagen deposition [17]. Its stimulating effect on leukocytes and potent antibacterial properties make CHI a widely used biomaterial in wound healing, available in forms such as non-wovens, hydrogels, films, and sponges [16,17].

In addition, CHI-based hydrogels also showed promise for burn wound healing. CHI-honey-gelatin hydrogels achieved complete epidermal repair by day 12 and were non-toxic [18]. Keratin-CHI-zinc oxide nanocomposites facilitated 92% repair in two weeks with good tensile strength and antibacterial activity [19]. Chitosan derivatives, particularly in combination with PVA, promote skin regeneration via non-toxic electrospun nanofibers [20]. Quaternary-CHI exhibits high antibacterial and antifungal properties [21], and PVA/quaternary-CHI electrospun mats show promise as wound dressings due to their effective antibacterial properties [22].

Gentamicin (Gent) is an aminoglycoside antibiotic known for its effective antimicrobial activity, making it widely used in treating microbial infections, particularly burn wounds [23]. However, its ability to penetrate the deeper layers of skin is limited due to low systemic absorption, likely because of its cationic nature [24,25]. Consequently, Gent primarily exerts its effects on the superficial skin layer. Despite its efficacy, Gent can cause renal tubular necrosis and congestion [26], as well as harm intra-auricular lymphocytes, leading to nephrotoxicity and ototoxicity, which somewhat restricts its clinical use [27]. To mitigate these toxic effects, researchers have explored methods to control Gent release by embedding or fixing it, allowing for sustained action [28]. Alternatively, Gent can be delivered directly to the target site for specific binding, enhancing its prolonged effect [29].

This study evaluated the in vivo burn wound healing potential of novel PVA/Gent and PVA/CHI/Gent hydrogels in a rat model. These hydrogels, originally synthesized in our laboratory using a green method with readily available components (PVA, CHI, and a low concentration of Gent), aim to provide inexpensive and commercially accessible wound dressings for sustained, local Gent release, thereby avoiding systemic antibiotic administration.

## 2. Materials and Methods

### 2.1. Materials

The following chemicals were utilized for the preparation of PVA/Gent and PVA/CHI/Gent hydrogels: PVA powder (fully hydrolyzed, Mw = 70–100 kDa, Sigma Aldrich, St. Louis, MO, USA), CHI powder (Mw = 190–310 kDa, deacetylation degree 75–85%, Sigma Aldrich, USA), and Gent sulfate solution (50 mg/mL in dH_2_O, Sigma Aldrich, USA). Deionized water was obtained by passing the distilled water through a GenPure ultrapure water system (TKA, Stuttgart, Germany).

#### 2.1.1. Synthesis of PVA/Gent Hydrogel

Colloid dispersion of PVA (10 wt.%) was prepared by dissolving PVA powder in hot distilled water at 90 °C for 2 h under magnetic stirring. The PVA hydrogels were obtained by physical cross-linking of PVA dispersion using the freezing-thawing method in 5 cycles. One cycle consisted of freezing for 16 h at −18 °C, followed by thawing for 8 h at 4 °C. Thus, the obtained hydrogels were cut into discs with diameters, *d*, of 10 mm and thicknesses, *δ*, of 4 mm. Then, the hydrogels were swollen in 5.0 mg/mL Gent solution at 37 °C for 48 h to obtain PVA/Gent hydrogels.

#### 2.1.2. Synthesis of PVA/CHI/Gent Hydrogel

A 10 wt% PVA solution was prepared by dissolving PVA powder in distilled water at 90 °C under magnetic stirring for 2 h. Separately, CHI was dissolved in 2 vol% acetic acid (CH_3_COOH) at room temperature under continuous stirring to obtain a 0.5 wt% solution. After cooling the PVA solution to room temperature, the CHI solution was added dropwise under stirring. The resulting PVA/CHI dispersion was homogenized by stirring at room temperature for an additional 2–3 h. Hydrogels were formed by physical cross-linking of the dispersion using a freeze–thaw technique. The mixture was subjected to five freeze–thaw cycles, each consisting of freezing at −18 °C for 16 h followed by thawing at 4 °C for 8 h. To obtain Gent-loaded hydrogels, the prepared hydrogels were immersed in a 5.0 mg/mL Gent solution and incubated at 37 °C for 48 h to allow drug absorption.

#### 2.1.3. Antibacterial Assays

Antibacterial activity of PVA/Gent and PVA/CHI/Gent hydrogels was investigated against Gram-negative (*Escherichia coli* ATCC 25922) and Gram-positive (*Staphylococcus aureus* TL) bacteria strains (culture collection Faculty of Technology and Metallurgy, University of Belgrade, Serbia). Hydrogels were sterilized in the laminar airflow chamber by exposure to a UV-C lamp (30 min, 60 cm lamp distance), and their antibacterial activity was evaluated by the disc-diffusion method. Soft agar (0.7 wt%, 15 mL) was melted and cooled to approximately 55 °C, then inoculated with 150 μL of overnight bacterial culture (~18 h incubation). The mixture was gently stirred to ensure uniform distribution and immediately poured over a pre-solidified nutrient agar base in sterile Petri dishes. The number of bacteria in the nutrient soft-top agar layer was set to be ∼10^6^ CFU mL^−1^. After solidification of soft-top agar, hydrogel disc samples were placed on its surface. The inhibition zone diameters were measured after 24 h incubation at 37 °C.

#### 2.1.4. Field-Emission Scanning Electron Microscopy (FE-SEM)

FE–SEM micrographs were obtained using Mira3 XMU FEG-SEM (Tescan, Brno, Czech), operated at 7 kV, with an SE detector.

#### 2.1.5. Equilibrium Swelling Degree

To determine the equilibrium swelling degree of PVA/CHI and PVA/CHI/Gent, hydrogels were dried until a constant mass was reached. Thus, the obtained dry xerogels were weighed and immersed in phosphate buffer (PB) containing K_2_HPO_4_ and KH_2_PO_4_ (pH∼7.4) and kept in a sterilizer oven at 37 °C. The weight of the swollen gels was measured periodically until an equilibrium swollen state was reached. At different times (every 1 h for the first 10 h, and after that at 24, 27, 30, 48, 50, 52, and 72 h), gels were taken out of the solution and weighed (the excess liquid from the surface was removed by wiping with dry paper). The equilibrium degree of swelling, *q_eq_*, was determined using the following Equation (1):

(1) $$q_{t}=\frac{m_{eq}-m_{0}}{m_{0}}$$

Here, *m_eq_* is the mass of the hydrogel at equilibrium swollen state, and *m*_0_ is the initial mass of the dry xerogel. All swelling measurements were carried out in triplicate.

#### 2.1.6. Gentamicin Release

High-performance liquid chromatography HPLC (Thermo Fisher Scientific, Waltham, MA, USA) was utilized for Gent component separation, and the detection and quantitative analysis were carried out in an ion trap mass spectrometer (MS) (LCQ Advantage, Thermo Fisher Scientific). The HPLC instrument was equipped with a reverse-phase column (4.6 mm × 75 mm × 3.5 μm) Zorbax Eclipse^®^ XDB-C18 (Agilent Technologies, Santa Clara, CA, USA), in front of which a precolumn (4.6 mm × 12.5 mm × 5 μm) was placed. The mobile phase consisted of methanol (A), deionized water (B), and 10% acetic acid (C). The optimized HPLC-MS operating parameters—including mobile-phase gradient; selected precursor ions; fragmentation transitions for quantification; and collision energies—were reported previously [30]. Mass spectra of Gent were acquired in the *m*/*z* range of 50–1000. As expected, the MS spectrum revealed three predominant ions corresponding to the major Gent components: Gent C1a, C2, and C1. These ions were selected as precursor ions, and their most sensitive transitions were used for quantitative analysis. Reported Gent concentrations represent the cumulative sum of the three identified components.

### 2.2. Animal Trials

Three-month-old male Wistar rats weighing 300–330 g obtained from the Department of Laboratory and Experimental Care and Use of Animals Unit of the Institute of Medical Research, Military Medical Academy (Belgrade, Serbia) were used in the experiment. Rats were housed individually in polypropylene cages in an air-conditioned animal facility under standard conditions with a photoperiodic cycle of 12 h light: 12 h darkness and food and water intake ad libitum. All of the experimental procedures were approved by the Ethical Committee of the Faculty of Veterinary Medicine, University of Belgrade, and by the Ministry of Agriculture, Forestry, and Water Management—Veterinary Administration (decision number 323-07-04903/2022-05/1). The experiment and animal handling were carried out in accordance with the ARRIVE guidelines and the European Union’s Directive 2010/63/EU on the Protection of Animals Used for Scientific Purposes.

#### 2.2.1. Experimental Design

Burn wounds were modeled following the protocol by Tavares Pereira et al. [31]. The procedure utilized general anesthesia with intraperitoneal injections of 75 mg/kg ketamine hydrochloride (Ketamidor 10%, 100 mg/mL, RICHTER PHARMA AG, Wels, Austria) and 10 mg/kg xylazine (Xylased 2%, BIOVETA, Ivanovice na Hané, Czech Republic). After trichotomy of the back skin and disinfection with povidone-iodine, a thermal injury was induced by applying a solid aluminum bar (10 mm diameter, mass of 51 g), previously heated in boiling water (temperature of 100 °C), to the skin of the dorsal proximal region for 15 s without any additional pressure. Post-procedure, animals received intramuscular analgesia with 5 mg/kg Ketoprofen (Ketonal, á 100 mg/2 mL, SANDOZ, Basel, Switzerland) for three consecutive days.

Following the burns, animals were randomly assigned to three experimental groups: control (Ctr) (*n* = 16), PVA/Gent treated (*n* = 16), and PVA/CHI/Gent treated group (*n* = 16), with daily dressing of the wounds. Clinical evaluations of the burn wounds were conducted on the 3rd, 7th, 14th, and 21st days by semi-quantitatively assessing the parameters such as blistering, edema, redness, crust, bleeding, secretion, and scar tissue on a scale from 0 (absent) to 3 (severe). Diameters of the burn were measured immediately after burn induction and on previously specified days to calculate wound contraction using Equation (2):

(2) $$X_{\%}=\left[X_{0}-X/X_{0}\right]*100$$

Here, *X*_%_ is the percentage of wound contraction on the specified day. *X*_0_ is the initial diameter of the wound, and *X* is the diameter of the wound on the specified day. On those evaluation days, four animals from each group were euthanized using 100 mg/kg Euthasol Euthanasia Solution (Produlab Pharma Production B.V., Raamsdonksveer, The Netherlands) for histological sample collection.

#### 2.2.2. Histological Analysis

During sampling, a square piece of skin containing the entire burn wound was taken and bisected along the diameter of the circular burn. Both halves of each sample were fixed and processed in a conventional manner for histological examination. Paraffin-embedded tissue blocks were serially sectioned and stained with Hematoxylin-Eosin (H/E) and Masson Goldner (MG) staining kits (Merck Millipore, Darmstadt, Germany), then evaluated on a standard Olympus CX31 microscope equipped with a digital camera and software (UC50 Soft Imaging Solutions camera and SensEntry 1.13 software, Münster, Germany). Histological analyses included the assessments of re-epithelization at the 14th and 21st days and evaluations of the dermis at all time points.

The re-epithelization process was evaluated on H/E-stained sections at 4× magnification by calculating the percentage of re-epithelization *X_e_*_%_ on the specified day using Equation (3):

(3) $$X_{e\%}=\left(X_{e}/X_{e}+X\right)*100$$

where *X_e_* is the length of newly formed epithelium on the specified day and *X* is the length of the open wound on the specified day. Additionally, the thickness of the newly formed epithelium was measured at five points approximately 300 µm from the wound edges on both sides of the burn wound at 40× magnification, and the stratification in the epithelium was assessed.

The semi-quantitative assessment of the dermis evaluated the percentage of dermal coverage by subepithelial neutrophils, fibroblasts, collagen deposition, edema, and angiogenesis at 40× magnification. Each parameter was scored as absent (0) for up to 10%, mild (1) for 10–40%, moderate (2) for 40–70%, and severe (3) for 70–100% coverage. All parameters were assessed on H/E-stained sections, except collagen deposition, which was estimated on MG-stained sections.

### 2.3. Statistical Analysis

All collected data were analyzed in the GraphPad Prism 9 software (GraphPad, San Diego, CA, USA). Prior to the application of the statistical analysis, the normality of data distribution was tested using the Shapiro–Wilk normality test. All histological examination results were analyzed using the Kruskal–Wallis test followed by Dunn’s post-hoc test, except for the re-epithelization assessment on the 14th day, which was analyzed using one-way ANOVA, followed by Tukey’s post-hoc test.

## 3. Results and Discussion

### 3.1. Antibacterial Activity

Before conducting in vivo experiments, the antibacterial activity of synthesized hydrogels with different combinations of substances (PVA, CHI, and Gent) was examined.

**Figure 1:**
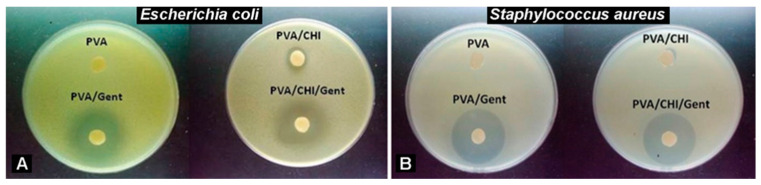
(**A**) Disc-diffusion test for *E. coli*; (**B**) *S. aureus* in the presence of hydrogels.

Figure 1 represents photographs of disc-diffusion tests against *E. coli* and *S. aureus* of PVA, PVA/Gent, PVA/CHI, and PVA/CHI/Gent hydrogels. All data are expressed as mean ± SD from three independent experiments.

The hydrogel comprised of PVA did not exhibit any antibacterial effect on the tested bacterial strains. Evidently, all the samples with CHI showed weak antibacterial activity, which was stronger against *E. coli* than *S. aureus*. The average width of the inhibition zone against *S. aureus* was 12.0 ± 0.2 mm. In the case of *E. coli*, two distinct regions of sensitivity are observed: a highly sensitive zone of 11.7 ± 0.3 mm and a moderate sensitivity zone of 12.8 ± 0.6 mm in diameter. As expected, the zones of inhibition are clearly visible for all Gent-containing hydrogels. Hydrogel PVA/Gent had a pronounced highly sensitive zone of 24.5 ± 0.4 mm and a moderate sensitivity zone of 34 ± 1.4 mm in diameter when tested against *E. coli*. The same sample acted very efficiently against *S. aureus*, since the inhibition zone was 31.5 ± 0.2 mm in diameter. In the case of PVA/CHI/Gent, the addition of antibiotic increased the antibacterial activity of hydrogels. Against *S. aureus,* PVA/CHI/Gent gave a 31 ± 0.2 mm wide zone of inhibition. When tested against *E. coli* difference in bacterial sensitivity was observed with a lighter-colored, highly sensitive zone of 26.9 ± 1.6 mm and a darker-colored, moderately sensitive zone of 30.1 ± 1.3 mm in diameter (Figure 1A,B).

The results obtained by the disc-diffusion test are in accordance with the kinetics of antibacterial activity against *E. coli* ATCC25922 and *S. aureus* found by Mišković-Stanković et al. [32]. Against Gram-negative *E. coli*, both PVA/Gent and PVA/CHI/Gent hydrogels demonstrated a strong bactericidal effect, reducing viable colony counts by over three orders of magnitude within 15 min of incubation. Complete sterilization was achieved after 1 h, with no detectable live *E. coli* cells. In contrast, for Gram-positive *S. aureus*, no viable cells were detected as early as 15 min post-inoculation. The mechanisms of antibacterial activity can be explained as follows. Gentamicin exerts its antibacterial effect by irreversibly binding to the 30S ribosomal subunit and 16S rRNA in bacterial cells. This interaction disrupts tRNA recognition, leading to misreading of mRNA and preventing the synthesis of essential proteins. Specifically, Gent binds to four nucleotides of 16S rRNA and an amino acid in the S12 protein, interfering with the ribosomal decoding site. As a result, incorrect amino acids are incorporated into the growing polypeptide chain, ultimately disrupting protein function and bacterial survival [33].

### 3.2. Characterization of Synthesized Hydrogels

**Figure 2:**
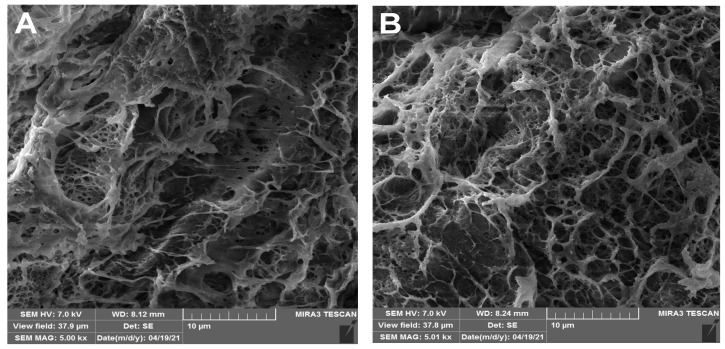
(**A**) SEM micrographs of PVA/Gent; (**B**) PVA/CHI/Gent hydrogels.

Scanning Electron Microscopy (SEM) images of PVA/Gent (Figure 2A) and PVA/CHI/Gent hydrogels (Figure 2B) revealed well-defined three-dimensional network structures characterized by uniformly distributed interconnected micropores. These observations suggest that the incorporation of the antibiotic does not affect the structural integrity of the hydrogels, thereby confirming its homogeneous distribution within the polymer matrix.

Interconnected porosity is a critical and desirable feature in the design of biomaterials, as it enhances the incorporation and sustained release of antimicrobial and antibacterial agents [34,35]. Moreover, such a porous architecture supports the efficient diffusion of essential nutrients and oxygen, which is vital for maintaining cellular viability and promoting tissue regeneration in biomedical applications [36,37]. In our previously published work, we thoroughly examined the nature of hydrogel formation by analyzing spectra of PVA, PVA/CHI, and PVA/CHI/Gent. The shift in the characteristic O–H stretching vibration band observed in the FTIR spectra of the PVA/CHI and PVA/CHI/Gent hydrogels suggests that no chemical reaction occurred between the hydrogel components. Instead, the shift indicates interactions involving hydrogen bonding between the –OH groups in the PVA matrix and water molecules, as well as hydrogen bond cross-linking between PVA hydroxyl groups and the –NH₂ and –OH groups present in Gent and CHI [30]. In the same article, we also analyzed tensile tests for PVA, PVA/CHI, and PVA/CHI/Gent films. The incorporation of CHI into PVA films resulted in enhanced mechanical performance. The tensile strength increased by 13.1%, from 47.25 MPa for pure PVA to 53.43 MPa for PVA/CHI films [32]. Likewise, Young’s modulus exhibited an 18.0% increase, from 1886.87 MPa to 2223.67 MPa. A substantial improvement was also observed in the tensile strain at maximum stress (*ε*_m_), which increased by 83.1%, from 7.27% to 13.31%. As the area under the stress–strain curve corresponds to material toughness, these findings collectively indicate a marked improvement in the toughness of the PVA/CHI films. These enhancements are primarily attributed to the establishment of strong physical interactions and hydrogen bonding between the hydroxyl groups of PVA and the amino and hydroxyl groups of CHI [38,39]. Conversely, the addition of Gent to the PVA/CHI matrix led to deterioration in mechanical properties. The tensile strength decreased by 31.7%, from 53.43 MPa (PVA/CHI) to 36.50 MPa (PVA/CHI/Gent), and Young’s modulus was reduced by 11.4%, from 2223.67 MPa to 1969.73 MPa. Notably, *ε*_m_ decreased significantly, becoming approximately five times lower than that of the PVA/CHI films. The altered shape of the stress–strain curves further suggests increased brittleness of the films upon Gent incorporation.

### 3.3. Swelling Properties

The ability to swell is one of the most important properties of hydrogel as a wound material dressing. Hydrogels should have a high degree of swelling to allow efficient absorption of wound exudate and, at the same time, provide local wound moisture to prevent drying and sticking of the dressing to the wound. The sorption behavior of PVA/CHI/Gent hydrogel was monitored in PB, containing K_2_HPO_4_ and KH_2_PO_4_ (pH ~7.4) at 37 °C. The swelling of hydrophilic polymer gels is a complex process, especially in the case of polymers containing ionizable functional groups, such as amino groups on CHI chains. Experimental data can be fitted by several kinetic models to determine the sorption parameters, such as the diffusion coefficients of the solvent through the polymer network. One of the most widely used sorption models is Etters model, which is regarded to provide a satisfactory description for the sorption processes.

Etters model, described by Equation (4), where *D* is the diffusion coefficient determined from the Etters model; *a*, *b*, and *k* are the Etters constants [40]. The equilibrium swelling degree (*q_eq_*) is defined as the maximum swelling degree reached at equilibrium, *t* is the time at which the swelling degree (*q_t_*) is reached, and *δ* is the thickness of the sample.

(4) $$\frac{q_{t}}{q_{eq}}=1-exp{\left[-k{\left(\frac{D_{E}t}{\delta^{2}}\right)}^{a}\right]}^{\frac{1}{b}}$$

**Figure 3:**
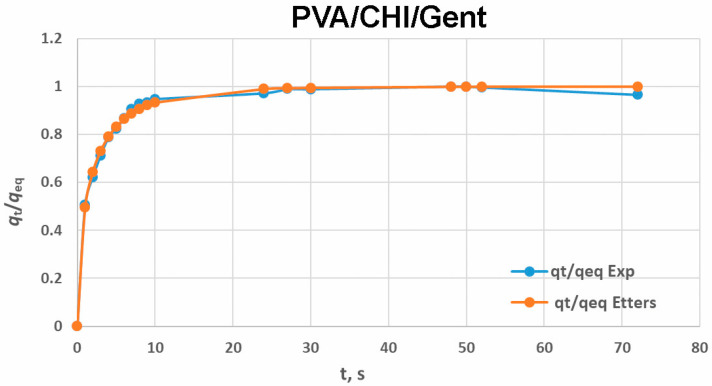
Etters approximations compared to the experimental data for PVA/CHI/Gent hydrogel.

Etters models (Figure 3) showed good agreement with the experimental profiles during the entire swelling period, which can be observed by the high value of the fit quality parameter (R^2^) given in Table 1. The diffusion coefficient was higher in hydrogel without Gent, which would mean that Gent does not have a favorable effect on swelling. On the other hand, *q_eq_* was higher in the hydrogel with Gent, contradicting the previous conclusion, as a higher *q_eq_*, indicates improved swelling capacity.

### 3.4. Gentamicin Release

To investigate the kinetics and mechanism of Gent release from PVA/Gent and PVA/CHI/Gent hydrogels, the experimental release profile of Gent has been determined using HPLC techniques, as the time dependence of the ratio *c_t_*/*c*_0_, where *c_t_* is the concentration of Gent released from the hydrogel at time, *t* and *c*_0_ is the initial concentration of Gent inside the hydrogel [32]. The experimental data were compared to several theoretical models in order to elucidate the Gent diffusion coefficient. The models applied were Makoid–Banakar [41], Korsmeyer–Peppas [42], and Kopcha [43], described by Equations (5)–(7), respectively:

(5) $$\frac{C_{t}}{C_{0}}=k_{MB}\cdot t^{n}\cdot exp(-c\cdot t)$$

(6) $$\frac{C_{t}}{C_{0}}=k_{KP}\cdot t^{n}$$

(7) $$\frac{C_{t}}{C_{0}}=A\cdot t^{1/2}+B\cdot t$$

where *k*_MB_ is the Makoid–Banakar constant; *c* is the Makoid–Banakar parameter; *k*_KP_ is the Korsmeyer–Peppas constant; *n*-the coefficient that describes the release transport mechanism (*n* < 0.5-Fickian diffusion, *n* > 0.5-non-Fickian/anomalous diffusion, *n* = 1-Case II transport) [42]; and *A* and *B*-Kopcha’s constants, which depend on the dominant transport phenomenon during release.

Drug release profiles from hydrogel matrices typically follow a common pattern characterized by an initial rapid release—known as the “burst release” effect—followed by a sustained; gradual release phase that eventually plateaus at approximately 80–100% release efficiency [44]. Several diffusion-based models that are most widely applied—Korsmeyer–Peppas; Makoid–Banakar; and Kopcha—offer advantages over classical models like Higuchi; Zero-order; First-order; Hixson–Crowell; and Baker–Lonsdale models; specifically for hydrogels. Classical models are less applicable to soft, swelling hydrogels (Hixson–Crowell) since they ignore matrix dynamics (First-order) or are good for spheres but not flexible for complex swelling/degradation (Baker–Lonsdale). The Korsmeyer–Peppas, Makoid–Banakar, and Kopcha models are better suited for hydrogels because they handle swelling, degradation, and polymer relaxation without requiring rigid assumptions about geometry or pure diffusion and therefore can describe complex, multi-phase release behaviors. Additionally, early-time approximations [45] are often employed to specifically describe the initial burst release phase.

Gentamicin release profiles verified the initial burst release effect of Gent from the hydrogel, i.e., 70% of the loaded antibiotic was released within the first 48 h, which could be very useful in preventing biofilm formation, followed by slow release of Gent in a later time period. The time exponent, *n*, provides insight into the dominant release mechanism. Since its values were below 0.5, the release of Gent from the hydrogel followed Fickian diffusion [42], indicating that drug transport was primarily driven by the concentration gradient. This conclusion was further supported by the Kopcha model, where the absolute values of parameter *A* (diffusion-controlled) were greater than those of *B* (erosion-controlled), confirming diffusion as the primary mechanism over polymer matrix relaxation. The calculated parameters for different models are listed in Table 2.

To estimate the diffusion coefficient of Gent, the Early-Time Approximation (ETA) model was employed. Two equations were applied: the standard ETA Equation (8) and a modified version proposed by Ritger and Peppas Equation (9) [45]. Ritger and Peppas emphasized that the standard ETA is often misapplied, as it is valid only under specific swelling conditions and for thin films with high aspect ratios (typically ~100), whereas the hydrogels used in this study—being thicker discs—exhibit aspect ratios closer to unity. In these Eqs, *c_t_*/*c*_0_ denotes the fraction of released Gent at the time *t*, *D* is the diffusion coefficient of Gent during the release, *t* is the time of release, *δ* is the hydrogel thickness, and *r* is the radius of the hydrogel disc:

(8) $$\frac{c_{t}}{c_{0}}=4\cdot {\left(\frac{D\cdot t}{\pi \cdot \delta^{2}}\right)}^{1/2}$$

$$\frac{c_{t}}{c_{0}}=4\cdot {\left(\frac{D\cdot t}{\pi \cdot r^{2}}\right)}^{1/2}-\pi \cdot \left(\frac{D\cdot t}{\pi \cdot r^{2}}\right)-\frac{\pi }{3}\cdot {\left(\frac{D\cdot t}{\pi \cdot r^{2}}\right)}^{3/2}+4\cdot {\left(\frac{D\cdot t}{\pi \cdot \delta^{2}}\right)}^{1/2}$$

(9) $$-\frac{2r}{\delta }\cdot \left[8\cdot \left(\frac{D\cdot t}{\pi \cdot r^{2}}\right)-2\pi \cdot {\left(\frac{D\cdot t}{\pi \cdot r^{2}}\right)}^{3/2}-\frac{2\pi }{3}\cdot {\left(\frac{D\cdot t}{\pi \cdot r^{2}}\right)}^{2}\right]$$

The ETA models represent the dependence of the fraction of released Gent on the square root of the release time, while the diffusion coefficient (*D*) of Gent release was determined from the slope of the initial linear part of the experimental curve. Using the modified ETA model, the value of *D* was calculated to be 7.16 × 10^−8^ cm^2^ s^−1^ for PVA/Gent hydrogel and 4.29 × 10^−8^ cm^2^ s^−1^ for PVA/CHI hydrogel, meaning that the release of Gent is slower from the hydrogel with CHI due to a greater number of bonds and consequently more crosslinked polymer matrix [46].

### 3.5. In Vivo Testing

The PVA/Gent and PVA/CHI/Gent hydrogels have been characterized, based on the material cytotoxicity scale [47], as non-toxic and suitable for in vivo testing [32]. Tests conducted on MTT assays even showed that PVA/CHI/Gent samples promote differentiation and growth of MRC-5 (human fibroblasts), as evidenced by cell viability values higher than 100%. However, in the case of L929 cells, the viability was slightly decreased for all samples, which could be due to the enhanced sensitivity of the mouse cell line toward our samples. These results are promising, as the main concern with biomaterials revolves around their immediate application and potential impact on surrounding tissue.

The in vivo analysis aimed to capture all phases of wound repair—inflammation; proliferation; and remodeling—by analyzing the wounds on the 3rd; 7th; 14th; and 21st days post-injury [31]. Inflammation begins with vascular responses and neutrophil infiltration, later transitioning to macrophage dominance, leading to the proliferative phase, which involves the formation of epithelium and granulation tissue, consisting of fibroblasts, collagen, and new blood vessels [48]. Semi-quantitative scales were used to assess healing parameters, reflecting standard practices in the literature [49,50,51,52].

### 3.6. Clinical Evaluations of the Burn Wound and Wound Contraction

Throughout the evaluation of PVA/Gent and PVA/CHI/Gent wound-healing efficacy on second-degree burns in Wistar rats, the tested hydrogels demonstrated good handling properties and hydrophilicity, allowing easy adherence to the wound bed. During the dressing changes for measurement and photography, the newly formed tissue remained intact, and the hydrogels did not adhere to the wound.

**Figure 4:**
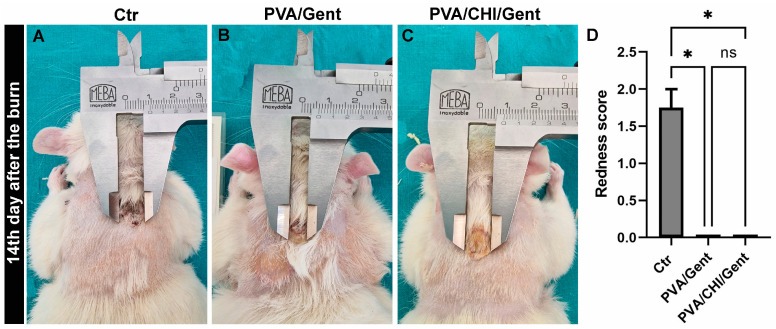
Representative photographs of burn wounds at the 14th day period from the (**A**) Control (Ctr); (**B**) Poly(vinyl alcohol)/Gentamicin (PVA/Gent) treated group; (**C**) Poly(vinyl alcohol)/Chitosan/Gentamicin (PVA/CHI/Gent) treated group; (**D**) boxplot showcasing redness scores compared by Kruskal–Wallis test + Dunn’s test. Boxes indicate the lower to upper quartile (25th–75th percentile) and median value. Whiskers extend to minimum and maximum values. Statistical significance is denoted as * *p* < 0.05.

Both hydrogels demonstrated enhanced healing relative to the Ctr group, showing reductions in all clinical parameters (blistering, edema, redness, crust, bleeding, secretion, and scar tissue), notably a significant decrease in redness by day 14 (*p* < 0.05) (Figure 4A–D). Considering that redness (*rubor*) was defined as one of the cardinal signs of inflammation 2000 years ago [53], its reduction confirms that both hydrogels reduce inflammation, which is also in accordance with the pronounced antibacterial activity of hydrogels containing Gent, shown in Section 3.1. In order to ensure inter-rater reliability in the semi-quantitative assessment of clinical parameters, the scoring was conducted by three independent researchers. Firstly, the researcher trained the scoring together by looking at the examples of scoring from the literature. The assessments were carried out in a double-blind method, with every researcher not knowing the scores given by others, and with assessed animals being encrypted by a fourth person to avoid biases.

**Figure 5:**
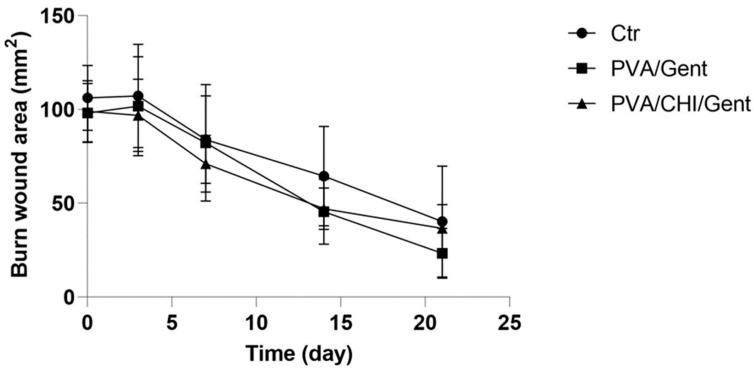
The graph depicting the wound area over time in the Ctr (line with circle), PVA/Gent-treated (line with square), and PVA/CHI/Gent-treated groups (line with triangle).

Wound contraction, a key indicator of healing, was monitored as an important part of the healing process of full-thickness wounds both in humans and in rats [12]. By the 3rd day period, the wound area slightly increased in both the Ctr and PVA/Gent groups compared to the initial measurement, while it decreased to a certain extent in the PVA/CHI/Gent-treated group, potentially due to CHIs ability to stimulate angiogenesis and fibroblast growth [18,54]. The property of CHI to inhibit the extension of burn wounds in the period of three days post-induction is described in the literature [55]. Throughout all other time periods, a reduction in wound area compared to the initial measurements was observed in all groups. While no significant differences were observed among groups by the study’s end, both PVA/Gent and PVA/CHI/Gent groups consistently exhibited smaller wound areas than the Ctr group, indicating that both hydrogels promote wound repair. This was most pronounced on the 14th day, with the Ctr group’s wound area measuring 64.47 ± 26.51 mm^2^, compared to 45.55 ± 17.33 mm^2^ in the PVA/Gent group and 47.41 ± 11.03 mm^2^ in the PVA/CHI/Gent-treated group (results are presented as mean ± SD) (Figure 5).

### 3.7. Histological Analysis

The histological analysis showed that the effects of PVA/Gent and PVA/CHI/Gent hydrogels on re-epithelization and healing processes in the dermis were not uniform and were significant in different time periods.

**Figure 6:**
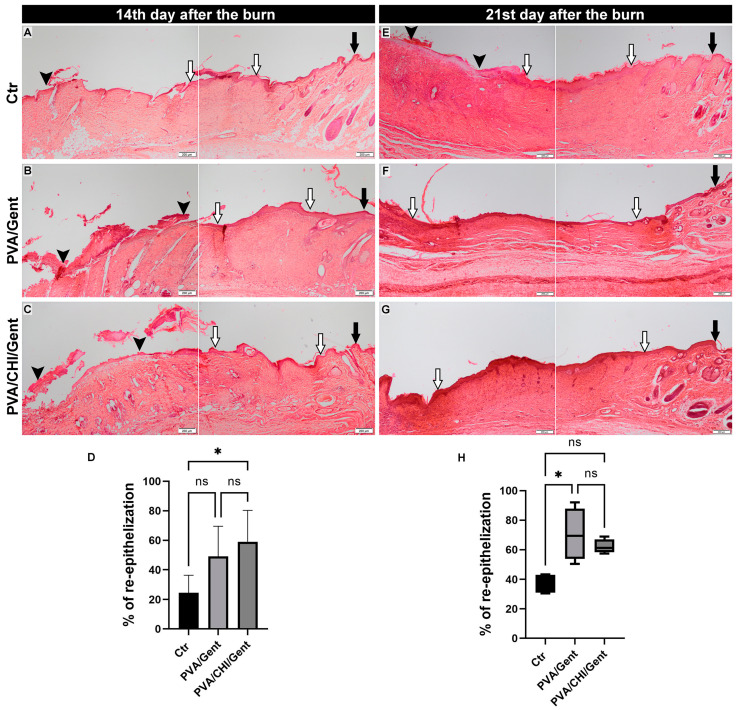
Representative photomicrographs of the burn wounds from the (**A**,**E**) Ctr group; (**B**,**F**) PVA/Gent treated group; (**C**,**G**) PVA/CHI/Gent treated group (black arrow—intact skin; white arrow—newly formed epithelium; black arrowhead–crust); (**A**–**G**) Tissue sections stained with hematoxylin/eosin and viewed with a scanning (4×), objective (bar: 200 µm); (**D**) Bar graph showcasing the percentage of re-epithelization at the 14th day period compared by the one-way ANOVA + Tukey test with data presented as mean ± standard deviation; (**H**) Boxplot showcasing the percentage of re-epithelization at the 21st day period compared by Kruskal–Wallis test + Dunn’s test. Boxes indicate the lower to upper quartile (25th–75th percentile) and median value. Whiskers extend to minimum and maximum values. Statistical significance is denoted as * *p* < 0.05.

Both hydrogels caused improvement of the re-epithelization process, which is depicted by representative microphotographs and graphs in Figure 6. On the 14th day, a significantly higher percentage of re-epithelization occurred only in the PVA/CHI/Gent-treated group compared to the Ctr group (*p* < 0.05) (Figure 6A–D). Crust formation was observed in all experimental groups (Figure 6A–C), with no significant differences in the thickness of the newly formed epithelium. In the Ctr group, some animals exhibited all epidermal strata, while others showed only the *stratum germinativum* or both the *stratum germinativum* and *stratum granulosum*. This pattern was also seen in the PVA/Gent group; however, animals in the PVA/CHI/Gent group showed the presence of all stratums in the newly formed epidermis. During the 21-day period, the higher percentage of re-epithelization was significant in the PVA/Gent-treated groups compared to the Ctr group (*p* < 0.05) (Figure 6E–H). The crust was present solely in the Ctr group (Figure 6E), and as in previous periods, there were no significant differences in the thickness of the newly formed epithelium among the experimental groups. By the 21st day, every animal in each group exhibited all strata in the newly formed epidermis. The relatively small sample size (n = 4 per group) in our study was determined based on the ethical considerations necessary for humane conduct of animal trials, represented by the principle of the 3Rs [56]. To ensure the robustness of statistical tests, precautions were taken prior to the experiment to control variation by keeping the animals in optimal conditions, as well as in the experimental design itself by practicing the principles of randomization and blinding [57].

Various studies examining the effectiveness of hydrogels containing different combinations of PVA, CHI, and Gent with or without other polymers showed beneficial effects on the process of re-epithelization [23,58,59]. To the best of our knowledge, this study is the first to explain the beneficial effects of the exact combination of PVA/Gent and PVA/CHI/Gent hydrogels. The enhanced re-epithelization could be explained by the fact that a moist environment enables the faster migration of keratinocytes [60]. The effects were evident earlier in the PVA/CHI/Gent-treated group, which can be explained by the synergy between the moist environment and CHI activating various cells (neutrophils, macrophages, and fibroblasts) to produce cytokines, which enables optimal conditions for keratinocyte migration [61].

**Figure 7:**
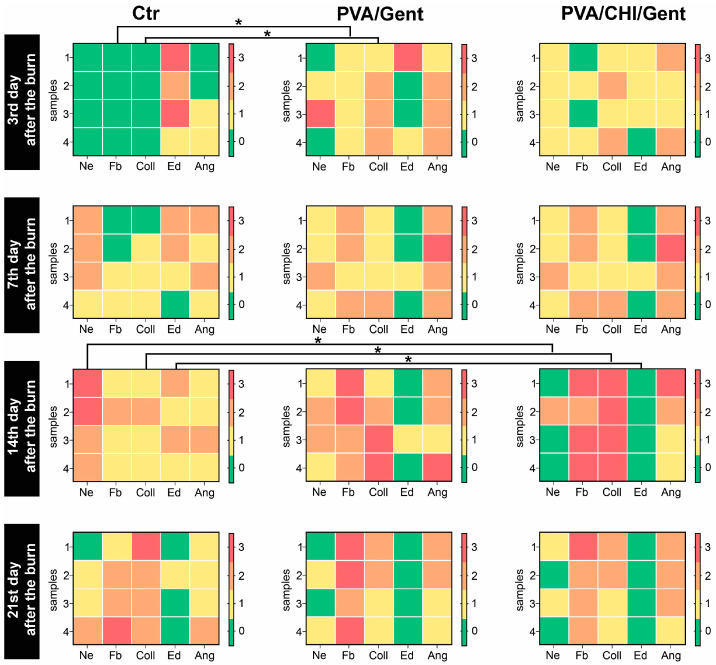
Heat maps showcasing the scores for all parameters [subepithelial neutrophils (Ne), fibroblasts (Fb), collagen deposition (Coll), edema (Ed), and angiogenesis (Ang)] assessed in the dermis of the burn wounds at 3rd, 7th, 14th, and 21st day periods in the Ctr, PVA/Gent, and PVA/CHI/Gent treated groups. The lines of significance are drawn between the columns representing the parameters that are significantly different between experimental groups. Statistical significance is denoted as * *p* < 0.05.

**Figure 8:**
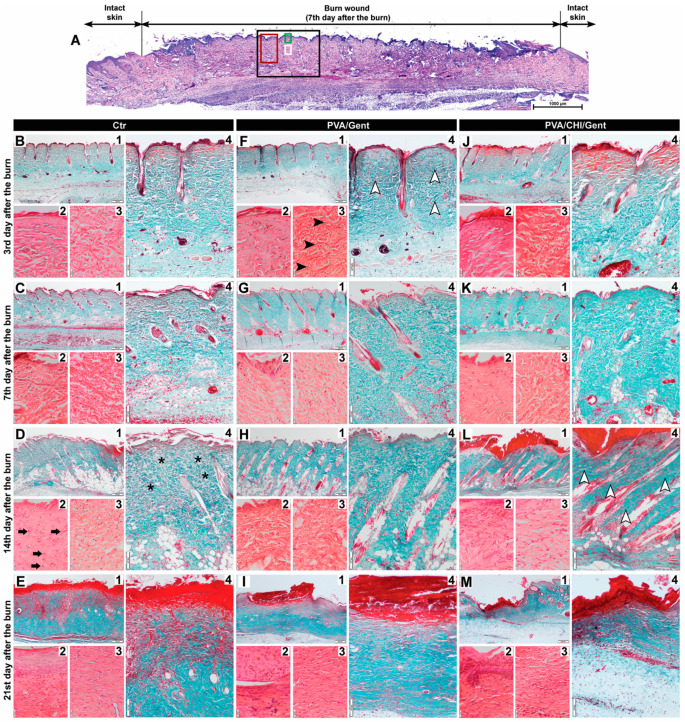
Photomicrographs of the burn wounds showcasing the parameters assessed in the dermis. (**A**) Composed photomicrograph illustrating the regions of the burn wound shown in other photomicrographs in this figure (black rectangle–region shown in photomicrographs 1; green rectangle–region shown in photomicrographs 2; white rectangle–region shown in photomicrographs 3; red rectangle–region shown in photomicrograph 4); (**B**–**E**) Burn wound sections of the Ctr; (**F**–**I**) PVA/Gent-treated group; (**J**–**M**) PVA/CHI/Gent-treated group (black arrowhead–fibroblasts; white arrowhead-collagen fibers; black arrow-subepithelial neutrophils; black star–edema). The parameters are marked in photomicrographs of groups in which there was a significant difference in scores of that parameter compared to other groups. Burn wound sections stained with Masson Goldner (**B1**–**M1**) viewed with a scanning (4×) objective (bar: 200 µm) and (**B4**–**M4**) low power (×10) objective (bar: 100 µm). Burn wound sections stained with Hematoxylin/Eosin (**B2**,**B3**,**C2**,**C3**,**D2**,**D3**,**E2**,**E3**,**F2**,**F3**,**G2**,**G3**,**H2**,**H3**,**I2**,**I3**,**J2**,**J3**,**K2**,**K3**,**L2**,**L3**,**M2**,**M3**) viewed with a high-power (×40) objective, (bar: 20 µm).

The individual scores for all parameters assessed in the dermis of the burn wound (subepithelial neutrophils, fibroblasts, collagen deposition, edema, and angiogenesis) are graphically represented in heat maps in Figure 7, while the representative photomicrographs of the assessments are presented in Figure 8.

At the 3rd day period, all parameters in the treated groups showed higher grades, except for edema, which was rated higher in the Ctr group (Figure 7). Significant differences were noted for fibroblast count and collagen deposition, with the PVA/Gent-treated group showing notably higher levels compared to the Ctr group (*p* < 0.05) (Figure 7). These findings suggest that the PVA/Gent hydrogel positively influences early wound healing by enhancing fibroblast proliferation and collagen synthesis. This observation aligns with in vitro studies where PVA hydrogels, even without added growth factors, induced slight increases in fibroblast proliferation and facilitated transit of cells [62]. The more pronounced increase in fibroblasts observed in our study may be attributed to the in vivo setting and the presence of different cells, namely neutrophils, during the inflammatory phase. Neutrophils protect the wound from infection and clear tissue debris [63], while also stimulating fibroblast proliferation both directly, via cytokine expression (IL-8, IL-1β, MCP-1), and indirectly by attracting macrophages, which further enhance fibroblast activity [64]. The significant increase in collagen deposition in the PVA/Gent group on the 3rd day is likely a direct consequence of the elevated fibroblast presence, considering the fact that the collagen is mostly synthesized by fibroblasts [63].

By the 7th day, edema remained more prominent in the Ctr group, which also exhibited higher grades of subepithelial neutrophils (Figure 7). Although fibroblast count, collagen deposition, and angiogenesis continued to be more pronounced in the treated groups, no significant differences were observed at this time point. This lack of significance may reflect the transitional phase of wound healing, where the inflammatory response begins to subside, and proliferative processes take precedence.

The trend persisted into the 14th day period, where the Ctr group had higher grades of subepithelial neutrophils and edema, with significant reductions in these parameters in the PVA/CHI/Gent group (*p* < 0.05) (Figure 7 and Figure 8D2,D4,L2,L4). Fibroblast count, collagen deposition, and angiogenesis remained more pronounced in the treated groups, with collagen deposition significantly higher in the PVA/CHI/Gent group (*p* < 0.05) (Figure 7 and Figure 8D4,L4). The delayed but significant effects of the PVA/CHI/Gent hydrogel likely result from the synergistic antimicrobial action of CHI and Gent. Chitosan’s antimicrobial activity, based on its positive charge interacting with negatively charged bacterial cell membranes, enhances permeability, while Gent’s limited cell penetration is overcome by CHIs effect [65,66]. Reduced subepithelial neutrophil presence and edema in the PVA/CHI/Gent group may thus reflect decreased inflammation due to the combined antibacterial action, which is consistent with our antibacterial activity tests and supported by literature [67]. Such enhanced antibacterial effects are desirable in wound dressings to prevent biofilm formation and bacterial adhesion during the critical early stages of healing. Also, the time-dependent release of the antibacterial agent would ensure prolonged sterility of the dressing and the wound itself. Moreover, the significant increase in collagen deposition at 14th day period in the PVA/CHI/Gent group aligns with previous findings where CHI stimulated collagen synthesis by enhancing prolyl hydroxylase activity in granulation tissue [68]. This enzyme’s peak activity at the 14th post-implantation day matches the elevated collagen levels observed in our study, reinforcing the efficacy of the PVA/CHI/Gent hydrogel in supporting the proliferative phase of wound healing.

By the 21st day, edema was no longer visible in any observed group (Figure 7). Although the Ctr group still exhibited more subepithelial neutrophils in the dermis, collagen deposition was rated higher in this group for the first time (Figure 7). Fibroblast presence and angiogenesis consistently remained higher in the treated groups, though none of the differences during this period reached significance. These results suggest that while initial inflammation is mitigated by the hydrogel treatments, long-term matrix remodeling processes like collagen deposition may vary depending on the wound environment and treatment dynamics.

## 4. Conclusions

The novel PVA/Gent and PVA/CHI/Gent hydrogels demonstrated significant wound-healing efficacy in second-degree burn wounds. The hydrogels showed good handling and hydrophilicity, adhering well to the wound bed. Both hydrogels had significantly reduced inflammation and exhibited strong antibacterial properties. They enhanced re-epithelization and increased fibroblast proliferation and collagen deposition in the healing process. Both PVA/Gent and PVA/CHI/Gent hydrogels show potential as effective wound dressings, promoting faster healing and improved tissue regeneration, showcasing that inexpensive and commercially available hydrogels can be efficiently used in burn wound treatment. Additional studies using molecular and immunohistochemical methods are a necessary next step in order to determine the exact mechanisms by which these novel hydrogels promote regeneration. After gaining a complete understanding of hydrogel functions on the molecular level, the hydrogels could be recommended for further clinical research.

## Figures and Tables

**Table 1:** Diffusion coefficients of the swelling medium, calculated from Etters sorption model, for PVA/CHI/Gent hydrogel.

Model		PVA/CHI/Gent
Etters	*D*_E_ (10^−6^ cm^2^ s^−1^)	1.41
	*a*	0.597
	*b*	0.0975
	*k*	0.681
	R^2^	0.998

**Table 2:** Parameters for different models of gentamicin release from PVA/CHI/Gent hydrogel.

Parameters	k_KP_ (s^−n^)	n	
Krosmeyer-Peppas model	0.4737	0.2137	
Parameters	k_MB_ (s^−n^)	n	C
Makoid-Banakar model	0.4956	0.2736	0.01972
Parameters	A (s^−1/2^)	B (s^−1^)	
Kopcha model	0.52356	−0.086433436	

## Data Availability

The data that support the findings of this study are available from the corresponding author upon reasonable request.
